# Immune checkpoint inhibitors in medulloblastoma: current updates in preclinical and clinical developments

**DOI:** 10.3389/fonc.2026.1725230

**Published:** 2026-05-08

**Authors:** Rachel L. Welch, Kirsten Moziak, Xingxing Zang, Allison M. Martin

**Affiliations:** 1 Department of Microbiology and Immunology, Albert Einstein College of Medicine, Bronx, NY, United States; 2 Marilyn and Stanley M. Katz Institute for Immunotherapy for Cancer and Inflammatory Disorders, Montefiore Einstein Comprehensive Cancer Center, Albert Einstein College of Medicine, Bronx, NY, United States; 3 Department of Pediatrics, Albert Einstein College of Medicine/Montefiore Medical Center, Bronx, NY, United States

**Keywords:** B7-H3, clinical trial, CTLA-4, immune checkpoint inhibitor, medulloblastoma, PD-1

## Abstract

Medulloblastoma (MB) is the most common pediatric embryonal tumor of the central nervous system. MB grows rapidly in the cerebellum and causes devastating disease in young children. The presence of high-risk molecular or histopathological features is associated with frequent recurrence, resistance to therapy, and poor prognosis, with few experiencing long-term survival. The current standard of care for MB includes surgical resection, radiation, and chemotherapy; however, there remains a need for more specific and effective treatment strategies, especially in recurrent disease. Promising developments in immune checkpoint inhibition have recently entered clinical trials, yet few address MB. Classic immune checkpoints such as Programmed Cell Death 1 (PD-1) and cytotoxic T lymphocyte antigen 4 (CTLA-4) have recently been identified as potential therapeutic targets for slowing MB progression in the recurrent setting. Preclinical and clinical studies have identified additional targets such as B7-H3, V-domain Ig Suppressor of T-cell Activation (VISTA), lymphocyte-activated gene 3 (LAG-3), P-selectin glycoprotein 1 (PSGL-1), and T cell immunoglobulin and mucin domain-containing protein 3 (TIM-3) expression in the MB TME. This review summarizes current preclinical and clinical prospects for immune checkpoint inhibition via direct antibody targeting for the treatment of MB and describes the proposed next generation of immune checkpoint inhibitors.

## 1 Introduction: medulloblastoma and the conundrum of the cold TME

Medulloblastoma (MB) is the most common pediatric embryonal tumor of the central nervous system (CNS) (1–4). MB tumors grow rapidly in the cerebellum and lead to cerebellar dysfunction, which manifests clinically as gait and coordination difficulties, headaches, nystagmus, nausea, and vomiting (5). Tumors are usually identified by neuroimaging of the brain using gadolinium-enhanced T1-weighted magnetic resonance imaging (MRI) and may involve the fourth ventricle or brainstem in addition to the cerebellum (1, 6–11).

The peak age at time of diagnosis is six to eight years, with median age at diagnosis of 7 years (1, 2). The incidence rate of MB is 10 times higher in children than in adults (1, 3), although MB may occur sporadically during adulthood. MB is the fourth most common pediatric CNS tumor, comprising 12.0% of pediatric CNS tumors, behind pilocytic astrocytoma, high grade glioma, and other glioma cases (2). The proportion of MB diagnosed annually is smaller than previously reported (4), suggesting that increased molecular characterization and diagnostic methods have allowed for more accurate classification of malignant brain tumors. MB continues to have a slight male predominance, wherein males are approximately 1.7 times more likely to be affected than females (2).

At the time of MB diagnosis, approximately one third of patients present with metastatic disease by leptomeningeal spread through cerebrospinal fluid and blood (12) and have high-risk disease with unfavorable rates of progression-free survival (PFS) (13–15). MB is fatal if left untreated and is among the leading causes of cancer-related mortality in children (5). Recent reports show that the 5-year relative survival for ages 0–19 years is 73.6%, and the infant age group has the worst survival rate of 56.6% (2). Although the most recent CBTRUS Report does not identify relative survival rates among MB risk strata or subgroups, 5-year survival rates for high-risk patients are below 70%, while standard risk patients have a survival rate between 70 and 90% (1, 9, 16).

Following diagnosis and subsequent surgical resection, histopathological separation of MB variants includes classic (as a small, round, blue cell tumor), desmoplastic/nodular, and anaplastic/large cell (11, 17, 18). Medulloblastoma with extensive nodularity is a unique group of desmoplastic/nodular tumors in infants with an excellent prognosis (11, 17, 18). More recently, epigenetic, transcriptomic, and genomic studies have revealed subgroup-specific molecular classification of MB tumors (19, 20), which reveals a need to develop subgroup-specific novel therapies (1, 21).

### 1.1 Medulloblastoma subgroup characterization

MB is comprised of four molecular subgroups, each classified by distinct histology and molecular features, in addition to prognosis and recurrence patterns. Group 1, the WNT-MB subgroup, which comprises only 10% of all MB cases, has the most favorable prognosis, with over 95% 5-year survival rates (1, 8). WNT-MB is characterized by somatic activating mutations in *CTNNB1*, which cause constitutively active WNT signaling by stabilizing β-catenin (8). In turn, β-catenin accumulates in the nucleus and drives upregulation of genes that promote cell growth and proliferation (22). The cell of origin for this subgroup derives from rhombic lip progenitor cells in the dorsal brainstem (1). Group 2, the SHH-MB subgroup, is most commonly characterized by loss-of-function mutations in *PTCH1* or *SUFU*, activating mutations in *SMO*, or amplifications in *GLI1/2* and *MYCN* (1). These mutations lead to ligand-independent activation of the SHH pathway, causing cell growth and proliferation in cerebellar granule cells (1). Unique to the SHH subgroup is the frequent presence of dominant negative *TP53* mutations at diagnosis. These patients represent about 20-30% of all SHH cases and fare especially poorly (23, 24). Recently, the presence of *MYCN* amplifications in SHH were shown to be associated with a very high-risk subset of SHH MB patients who have a PFS of only around 20% (25). While *MYC* amplifications are less common at diagnosis in SHH MB than *TP53* mutations, both individually confer a similarly poor survival (25).

Together, Group 3/4 (non-WNT/non-SHH) MB is the most common subgroup and is often considered as one group due to the difficulty in distinguishing Group 3 or Group 4 based on histopathology alone (19). Additional heterogeneity is currently being described to reveal subtypes within each subgroup (1, 8, 26). Group 3 MB occurs almost exclusively in infants and young children and is associated with the highest rate of metastasis at diagnosis and confers the worst survival outcomes for any subgroup (<60% 5-year survival) (8). Although Group 3 MB lacks a common driver pathway, it is thought to originate from neural stem populations (25). Alterations in *MYC* amplifications are observed in Group 3 and are associated with a poor prognosis (25). Group 4 MB is frequently metastatic at presentation and likely originates from unipolar brush cells (8). *MYC* amplifications are less common in Group 4 and, when present, have fewer effects on survival (25). Together, Group 3/4 MB recur at a higher rate than other subgroups.

Interestingly, regardless of initial subgroup*, MYC* alterations and dominant-negative *TP53* mutations, are the most commonly gained mutations in the setting of recurrent disease (27). *MYC* alterations and dominant-negative *TP53* mutations confer poor survival (1, 27, 28). Patients with refractory and recurrent disease among the Group 3/4 subgroup have a particularly poor prognosis, with survival rates less than 20% after recurrence (29).

### 1.2 Current practices for MB treatment: standard of care

Standard of care for treatment of standard-risk MB includes maximal safe surgical resection, craniospinal irradiation in children older than three years of age, and adjuvant cisplatinum-based cytotoxic chemotherapy (13, 30–32). Risks and toxicities from surgical resection include cerebellar mutism and memory deficits (33). Chemoradiotherapy frequently induces nausea/vomiting and myelosuppression, among other acute side effects (33). Secondary malignancy, fertility impairment, hearing impairment, neurocognitive deficits, and endocrine or cardiac dysfunction remain long-term risks of chemoradiotherapy treatment (33). Proton radiotherapy, an alternative radiotherapy to photon therapy, delivers highly-concentrated proton beam radiation to targeted areas of tumor and the cranio-spinal axis (30). Proton therapy is superior to photon therapy in its ability to spare surrounding healthy tissue and is now part of the standard of care therapy for MB (30, 34, 35). Treatment-related risks of secondary malignancy and neurocognitive or intellectual deficits are demonstrated to be reduced after proton therapy, but not eliminated (30, 34–36).

### 1.3 Immunosuppressive tumor microenvironment of MB

To date, no clinically effective immunotherapy exists for any individual subtype of MB (37). One major barrier to the development of immunomodulatory therapies for MB is the immunosuppressive, or cold, tumor microenvironment (TME). The cold TME in MB is characterized by few tumor-infiltrating lymphocytes, clusters of regulatory cells at the tumor border, and the presence of myeloid-derived suppressor cells to inhibit anti-tumor immunity (38–41). Dysfunctional chemokine gradients may also contribute to poor T-cell infiltration. For example, MB tumor cells may secrete chemokines such as CCL-2 to promote the recruitment of potentially immunosuppressive and pro-tumor monocyte-derived macrophages (42). The few T cells that do infiltrate the tumor are shown to be subsequently suppressed by both immunosuppressive cytokines such as Transforming Growth Factor Beta (TGF-b) and inhibitory immune checkpoints expressed on tumor cells or in the TME (43). The lack of T-cell-mediated anti-tumor immunity in MB has also been attributed to low mutational neoantigen load and ineffective antigen presentation (44, 45). However, recent studies reveal that MB may have a greater capacity for antigen presentation on both MHC-I and MHC-II than previously reported (46, 47), suggesting that if barriers to T-cell infiltration and activation were ameliorated, functional anti-tumor immune responses could be generated.

**Figure 1:**
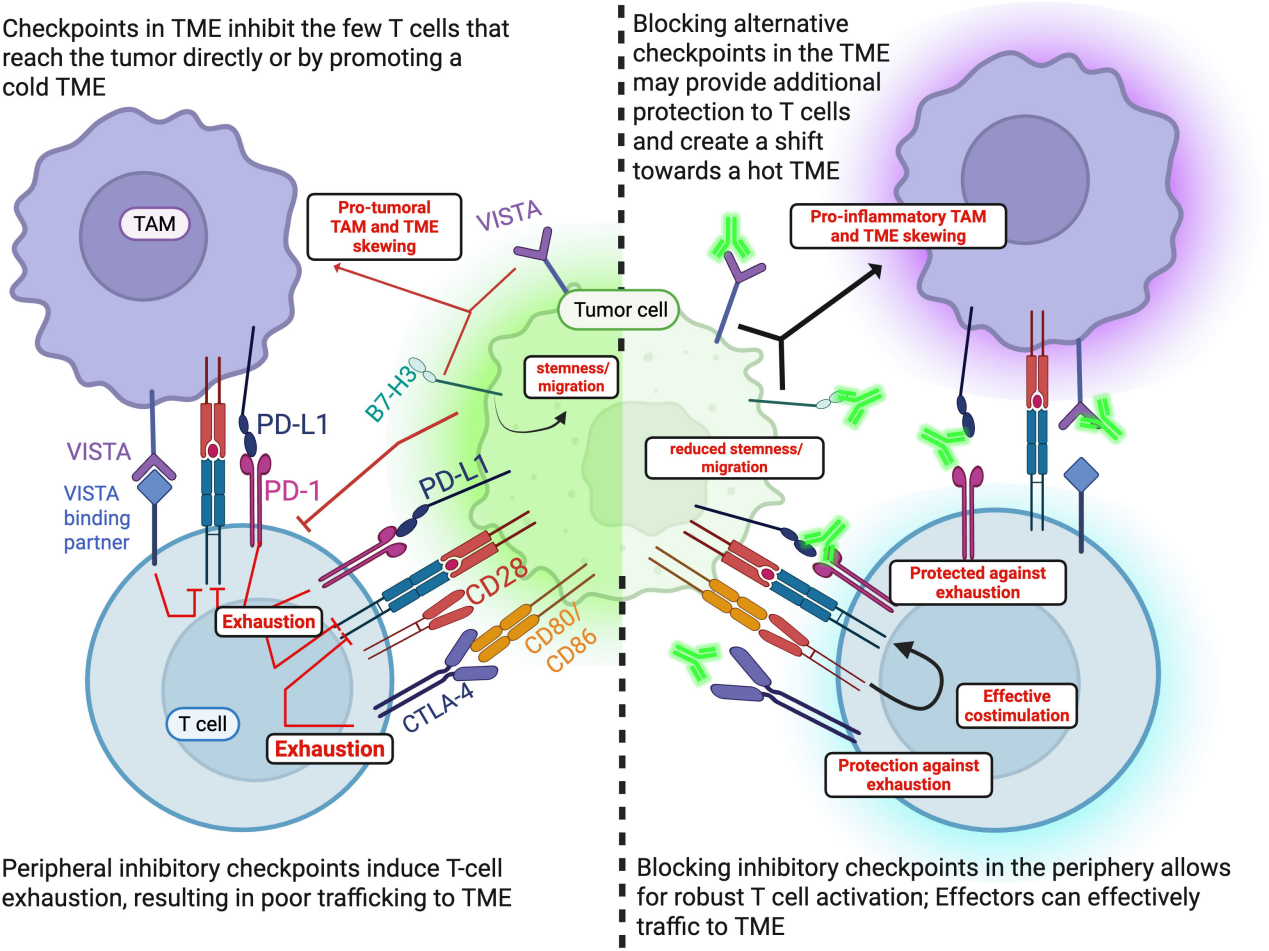
Summary of immune checkpoints and inhibitor targets. Made with Biorender.

Blocking inhibitory immune checkpoint molecules provides one opportunity whereby these obstacles have been overcome in other tumors. Classic examples of immune checkpoints include Programmed Cell Death-Ligand 1 (PD-L1), its binding partner Programmed Cell Death 1 (PD-1), and Cytotoxic T-Lymphocyte Associated protein 4 (CTLA-4). Alternative immune checkpoints, such as B7-H3 and V Domain Ig Suppressor of T-cell Activation (VISTA) are often present in cold TMEs such as in MB (39–41). This review summarizes the current immune checkpoint landscape in MB and discusses the prospects for targeted immunotherapy in the treatment of MB from the clinical and preclinical literature, as well as their limitations (**Figure 1**).

## 2 Preclinical prospects: immune checkpoints and immunotherapy in medulloblastoma

Natural antitumor immunity is mediated via activation of tumor-specific cytotoxic T cells, anti-tumor M1 macrophages, tumor antigen-presenting dendritic cells, and natural killer (NK) cells. The body’s natural antitumor immunity is restrained by a tumor’s immune evasive approaches, such as downregulating MHC-I expression, secreting immune suppressive cytokines, or exploiting inhibitory immune checkpoints (48). Under homeostatic conditions, immune checkpoints inhibit immune system activation to prevent autoimmunity and hyperactive immune responses (49). Some immune checkpoint proteins decrease proliferation and activation of T cells by downregulating T-cell receptor (TCR) signaling (50, 51). Other immune checkpoints alter cell cycle progression, gene transcription, and epigenetic programming to induce quiescence (52) or apoptosis (53). Novel immunotherapies, including immune checkpoint inhibitors and adoptive cellular therapies are currently in development to promote tumor clearance.

### 2.1 PD-1/PD-L1

The B7/CD28 family is one of the most prominent family of immune checkpoints and comprises both co-stimulatory and co-inhibitory molecules including PD-1/PD-L1 and B7-H3 (49). PD-1/PD-L1 are immunoregulatory molecules variably expressed in solid and central nervous system tumors (54). PD-1 is a type 1 transmembrane protein with an immunoreceptor tyrosine-based inhibitory motif (ITIM) and an immunoreceptor tyrosine-based switch motif (ITSM) in its cytoplasmic tail (55, 56). While PD-1 is expressed in both mouse and human B and T cells (57), PD-L1 or PD-L2 is often expressed on antigen presenting cells and regular host tissue (58) but can be expressed by cancer cells (59). Upon ligation between PD-1 and PD-L1/2, B-cell mediated antibody generation and cytotoxic T-cell mediated killing are suppressed (55, 60).

The PD-1/PD-L1 axis is among the most prominent clinically inhibited immune checkpoint pathways in current therapeutic settings. The overwhelming majority of studies have found MB cells to have low PD-L1 expression by both transcript and protein expression, and the few PD-1-expressing lymphocytes found in MB tumors are typically isolated to perivascular regions (61–63). However, some studies demonstrate that SHH- and WNT-MB have constitutively high gene expression of PD-L1 while Group 3/4 tumors rarely express PD-L1 (44, 62). Conflicting reports revealed that SHH- and WNT-MB may instead have undetectable PD-L1 expression by IHC (44, 62). Discordance of PD-L1 transcript with cell surface protein expression is well documented in cancer due to structural variations as well as posttranscriptional and post translational modifications (64, 65). Although these mechanisms have not been specifically tested in MB, studies that describe only transcript level expression should be interpreted with caution as this may not correlate with increased cell surface expression and function. Variation in detection methods between PD-L1 antibody clones has also been well described (66–71) and may contribute to conflicting findings in PD-L1 expression levels in MB. Induction of PD-L1 expression on MB cells has been observed in SHH and Group 3/4 cell lines in response to IFNγ and was also observed in patient-derived organoids as well as Group 3 and SHH-MB cell lines in response to NK cell confrontation (72). Inducing PD-L1 expression via IFNγ stimulation by immune modulation (62) or NK cell accumulation (72) may serve as a potential avenue to boost the efficacy of anti-PD-1 therapy.

It remains unclear whether PD-L1 expression on tumors cells is a positive indicator of response to targeted anti-PD-1 therapy in MB. For example, PD-L1 expression was not predictive of therapeutic response to anti-PD-1 treatment when comparing a syngeneic mouse model of SHH-driven MB, expressing higher PD-L1 *in vivo*, to a syngeneic Group 3 MB model, expressing low PD-L1 (73). Surprisingly, Group 3 tumor-bearing animals in this study had a higher baseline expression of PD-1+ CD8 T cells in the TME and a more effective response to PD-1 blockade (73). Peripheral responses may underlie increased efficacy of PD-1 blockade in Group 3 MB animals despite the lack of PD-L1 expression. For example, neither subgroup demonstrated effective anti-PD-1 monoclonal antibody binding to infiltrating lymphocytes in the TME, yet both tumor groups experienced increased PD-1-negative T cell influx (73). Further exploration is required to fully elucidate these mechanisms in MB.

### 2.2 CTLA-4

Cytotoxic T-lymphocyte associated protein 4 (CTLA-4, or CD152) transmits an inhibitory signal to T cells via competitive binding to CD28 (74). CTLA-4 is a transmembrane protein with an extracellular surface receptor that is similar to CD28 (74). Under homoeostatic conditions, CTLA-4 is contained in micro-vesicles in the cytoplasm of regulatory and anergic T cells, which are trafficked to the surface by T-cell interacting molecule (TRIM) when a T-cell receptor (TCR) becomes activated (75). In the absence of CTLA-4, CD28 binds to its ligand CD80 or CD86 to serve as a co-stimulatory signal in priming of naive T cells by an antigen presenting cell (75). CTLA-4 inhibits the interaction between CD28 and CD80/86 by directly binding CD80/86, thus inhibiting T-cell activation (75).

Cell surface CTLA-4 expression on CD-4+ T cells is low among SHH-MB and Group 3 MB syngeneic mouse models (73). Tumor-associated macrophages express myeloid-specific CTLA-4 in the TME of a syngeneic mouse model of MYC-driven MB and in human MB (41). Multiplex immunofluorescence staining of tissue microarrays (TMA) recently revealed that on the surface of MB tumor cells, CTLA-4 is expressed in both the WNT and Group 4 subgroups and higher in the WNT subgroup (76). Although these subgroups are generally associated with better prognosis, especially WNT, in the overall cohort, elevated CTLA-4 expression had a negative association with PFS (76). In a preclinical murine study, the use of anti-CTLA-4 blocking antibodies alone or in combination with anti-PD-1 blocking antibodies did not show a survival benefit for SHH-MB, but combination therapy did yield a survival benefit for Group 3 MB animals (73). However, neither Group 4 nor WNT have been evaluated this way. There are very few preclinical studies that explore the mechanistic role of CTLA-4 in the TME of MB and even fewer that elucidate the translational readiness of CTLA-4 inhibition in MB treatment at recurrence or among high-risk subgroups.

### 2.3 B7-H3

B7-H3 (CD276) is a type-1 transmembrane protein in the same B7 ligand family as PD-L1 (77). B7-H3 is most often expressed on tumor cells but has also been discovered on tumor-associated macrophages (TAMs) (78) and tumor stroma (79). Although high B7-H3 expression on tumor cells has consistently correlated with poor outcomes in MB patients (80, 81), there are conflicting reports of differential B7-H3 expression among subgroups (82–86). Publicly-available MB datasets evaluating the *B7-H3* mRNA expression between subtypes identified that *B7–H3* expression is highest in the WNT subtype and lowest in the SHH subtype (85). In a TMA by the same group, antibody staining revealed B7-H3 protein to be positive in all 33 samples evaluated. Only one sample was described as having “low” staining (85). Another TMA revealed that 96% of MB samples stained positive for B7-H3 by IHC, and Group 4 had the most frequent B7-H3 protein expression in this study (86). High B7-H3 expression in this TMA was also associated with lower CD-3 T-cell infiltration and lower γδ T-cell infiltration in the TME (86). Survival associations with B7-H3 in MB may be confounded by variations in epitope targeting with different antibody clones and scoring criteria used across studies in addition to disease specific factors including subgroup distribution and molecular features.

While the exact role of B7-H3 in MB is not yet fully understood; in cancer, emerging evidence suggests B7-H3 plays a role in disease progression and relapse/recurrence. For instance, microRNAs, such as miRNA-1253 and miRNA-29, may serve a tumor suppressor role in MB via B7-H3 inhibition (84, 87). MiRNA-29 was shown to be involved in directly and indirectly silencing B7-H3 in MB (84). MiRNA-29 has been shown to silence MYC and MYC in turn binds the B7-H3 promotor to directly drive expression (84). B7-H3 is thus of particular interest in the *MYC*-amplified Group 3 MB as well as in recurrent/refractory patients, given that *MYC* amplification is one of the most gained defects in patients at the time of recurrence (27). Furthermore, *MYC-*amplified Group 3 MBs overexpress EZH2, and EZH2 is associated with poor prognosis in MB (83). It has been demonstrated that inhibiting EZH2 in Group 3 MB cell lines reduces both *MYC* and B7-H3 expression and impairs cell viability (83). The authors concluded that in Group 3-MB, EZH2 promotes *MYC* expression, which represses MiRNA-29, resulting in increased B7-H3 expression and enhanced immune evasion, angiogenesis, and cancer stemness (83).

Preclinical therapeutics are currently being developed to leverage the high and specific expression of B7-H3 protein in MB via engineered immune cells, antibody drug conjugates (ADCs), and FC-enhanced antibodies. For instance, FC-enhanced antibody, MGA271 (enoblituzumab), has increased affinity for the activating receptors CD32A and CD16A on NK cells to mediate antibody-dependent cellular cytotoxicity (ADCC) (88). Enoblituzumab was also used to develop CAR-T cells for *in vivo* syngeneic SHH and Group 3/4 MB models, which demonstrated effective blood-brain-barrier penetrance into the CNS, robust effector responses, and increased survival (89). Additionally, bispecific αB7-H3-αCD3 Chemically Self-Assembled Nanorings (CSANs), which are made up of monomers with multivalent binding domains against either B7-H3 or CD3, have recently been developed (90). CSANs assemble into ring-like structures and, due to their bi-specificity towards a tumor associated antigen (B7-H3) and tumor cells, cluster T cells toward tumor cells to facilitate cytotoxicity (90). CSANs were also effective in entering the blood brain barrier in an immunodeficient MB mouse model (90).

Lastly, antibody drug conjugates (ADCs) have been developed to exploit the specificity of antibody-bound B7-H3 to deliver topoisomerase inhibitors to solid tumors (91) or to deliver radiation therapy to MB cells (NCT04167618, NCT04743661, NCT05064306) (92, 93). Intraventricular compartmental radioimmunotherapy (cRIT) is a novel immunotherapy that targets radioactive isotopes Iodine-131 or Lutetium-177 to tumor tissue to mediate tumor destruction and delay disease progression in MB and neuroblastoma (93–96). Omburtamab has recently been proposed as a candidate monoclonal antibody targeting B7-H3 and is currently under investigation in clinical trials of recurrent or refractory MB. One report proposed a phase I/II dose-escalation for intracerebroventricular radioimmunotherapy using 177-Lu-DTPA-omburtamab in pediatric patients with recurrent/refractory MB (92); while another described that 177-Lu-DTPA-omburtamab concentrated in CSF as expected when examining the biodistribution and radiation dosimetry after intrathecal delivery (97). A recent study sought to identify the predictors of response, patterns of failure, and radiologic events upon intraventricular 131-I-omburtamab treatment after external beam radiotherapy in patients with recurrent MB and ependymoma (93). Interestingly, survival was lower in the non-WNT/non-SHH cohort as compared to the SHH-MB patients in the study. Importantly, if patients had no evidence of disease (NED) at the time of cRIT initiation, this was associated with improved PFS and OS, and two patients achieved complete response.

## 3 Clinical prospects: immune checkpoint inhibition in medulloblastoma

Few clinical trials (n=7) address the use of immune checkpoint inhibitors for MB immunotherapy (**Table 1**). Instead, recent clinical trials for MB treatment largely focus on Sonic Hedgehog pathway antagonist therapy for SHH MB (101–104), radiolabeled monoclonal antibodies targeting non-checkpoint molecules (95, 105), and CAR T-cell therapy (106–110). To date, only seven clinical trials investigate the use of anti-PD-1 and anti-CTLA-4 targeted immunotherapy in pediatric and adult MB patients.

**Table 1:** Clinical trials for immune checkpoint inhibitors in treatment of medulloblastoma patients.

Title	National Clinical Trial (NCT) Number	Sponsor	Date of Most Recent Update	Trial Phase	Status	MB Inclusion Criteria	Treatment Regimen	Goals of Study for MB Patients	Publication Status	Year Published	First Author	Type of Article	Title
Pembrolizumab in Treating Younger Patients With Recurrent, Progressive, or Refractory High-Grade Gliomas, Diffuse Intrinsic Pontine Gliomas, Hypermutated Brain Tumors, Ependymoma or Medulloblastoma	NCT02359565	NCI	8/1/25	I	Active, not recruiting	Recurrent or refractory Pediatric	Anti PD-1 (Pembrolizumab) every 21 days for 24 cycles	Identify safety, adverse effect, survival, and response rate following administration of an adult dose of pembrolizumab in a pediatric population	Yes	2024	Hwang	Abstract	NOAE064.379 IMMU-08. Outcome of Patients with Recurrent Medulloblastoma and Ependymoma Treated with Pembrolizumab, an Immune Checkpoint Inhibitor: A Pediatric Brain Tumor Consortium Study (PBTC045) (98)
Immune Checkpoint Inhibitor Nivolumab in People With Recurrent Select Rare CNS Cancers	NCT03173950	NCI	6/29/25	II	Completed	Recurrent or refractory Adult	Anti PD-1 (Nivolumab), every 2 weeks for 1-2 cycles at low dose, every 4 weeks for 14 cycles at higher dose	Determine the efficacy and disease control rate for patients with recurrent, refractory medulloblastoma	Yes	2021	Penas-Prado	Abstract	CTIM-32. Immune Checkpoint Inhibitor Nivolumab in People with Recurrent Select Rare CNS Cancers: Results of Interim Analysis in a Heavily Pretreated Cohort (99)
A Study of Bempegaldesleukin (BEMPEG: NKTR-214) in Combination With Nivolumab in Children, Adolescents and Young Adults With Recurrent or Treatment-resistant Cancer (PIVOT IO 020)	NCT04730349	Bristol-Myers Squibb	3/24/23	I, II	Terminated	Recurrent or refractory Pediatric and young adult	Anti PD-1 (Nivolumab) and PEGylated IL-2 (NKTR-214/BEMPEG/Bempegaldesleukin)	Identify the safety, tolerability, efficacy, and drug levels Bempegaldesleukin (IL-2 agonist) with nivolumab in children, adolescents, and young adults	No				
Fourth Ventricular Administration of Immune Checkpoint Inhibitor (Nivolumab) and Methotrexate or 5-Azacytidine for Recurrent Medulloblastoma, Ependymoma, and Other CNS Malignancies	NCT06466798	The University of Texas Health Science Center, Houston	2/12/25	I	Recruiting	Recurrent Pediatric and adult	Anti PD-1 (Nivolumab) and methotrexate	To assess the safety, toxicity, and antitumor activity for 4th ventricle infusion of nivolumab and methotrexate	No				
PEP-CMV + Nivolumab for Newly Diagnosed Diffuse Midline Glioma/​High-grade Glioma and Recurrent Diffuse Midline Glioma/​High-grade Glioma, Medulloblastoma, and Ependymoma (PRiME II)	NCT06639607	Washington University School of Medicine	8/1/25	I, II	Not yet recruiting	Recurrent Pediatric and young adult	Anti PD-1 (Nivolumab), CMV-directed peptide vaccine, and temozolomide	Evaluate toxicity, change in immune response, overall survival, and progression-free survival with combination CMV-peptide vaccine and nivolumab with temozolamide	No				
Adoptive T Cell Therapy, DC Vaccines, and Hematopoietic Stem Cells Combined With Immune checkPOINT Blockade in Patients With Medulloblastoma (MATCHPOINT)	NCT06514898	University of Florida	7/8/25	I	Active, not recruiting	Recurrent or refractory Group 3/4 MB	DC vaccine and anti PD-1	A single-arm, unblinded, uncontrolled pilot study to evaluate the feasibility, toxicity, and safety of Adoptive T Cell Therapy and anti-PD-1 antibody in children and young adults with recurrent or progressive Group 3/4 MB after CSI radiation	No				
A Study to Evaluate the Safety and Efficacy of Nivolumab Monotherapy and Nivolumab in Combination With Ipilimumab in Pediatric Participants With High Grade Primary Central Nervous System (CNS) Malignancies (CheckMate 908)	NCT03130959	Bristol-Myers Squibb	8/9/22	Ib, II	Completed	Recurrent or refractory Pediatric	Anti PD-1 (Nivolumab) alone once every 2-weeks; or anti PD-1 (Nivolumab) with with anti-CTLA-4 (Ipilimumab) once every 3-weeks for 4 doses then Nivolumab once every 2-weeks	Evaluate the safety, dose-limiting toxicity, adverse events, overall survival, and progression-free survival of nivolumab alone or in combination with ipilimumab	Yes	2023	Dunkel	Full-Length Research Article	Nivolumab with or without ipilimumab in pediatric patients with high-grade CNS malignancies: Safety, Efficacy, biomarker, and pharmacokinetics - CheckMate 908 (100)

### 3.1 Anti-PD-1 immune checkpoint inhibition

Pembrolizumab (PB) and Nivolumab (NIVO) are humanized IgG monoclonal antibodies which target checkpoint molecule PD-1. PB and NIVO are FDA-approved to treat certain advanced cancers, such as non-small cell lung cancer and melanoma, as both monotherapy and combination therapy (111, 112). Clinical trials using anti-PD-1 blocking antibodies as a monotherapy for patients with recurrent or refractory MB, among other CNS malignancies, in children and adults, are summarized in **Table 1**. The goal of these clinical trials is to identify the safety, adverse effects, and disease control rate of PB or NIVO therapy in patients with recurrent and refractory MB. Only three of these studies have published their results to date.

A single-institution retrospective review was conducted by Rady Children’s Hospital to identify outcomes associated with NIVO treatment in patients with recurrent or refractory pediatric brain tumors, including one pediatric patient with MB (113). Prior to NIVO administration, the MB patient received radiation and chemotherapy, and the tumor was PD-L1 negative by IHC without specific subgroup identification. Nivolumab was given every 2 weeks, but the patient only received 3 treatments. Overall survival (OS), PFS, and safety profiles in this patient were not specified. The authors concluded from the study that NIVO should be limited to pediatric patients with elevated PD-L1 expression across pediatric brain tumor diagnoses, however these results are limited by the small sample size of one.

NCT03173950 evaluated the efficacy of NIVO treatment for recurrent CNS malignancies, including MB, in adult patients who have or have not received heavy pretreatment (114). Heavy pretreatment was defined as receiving more than 3 prior therapies, such as radiation, surgery, or chemotherapy (114). NIVO was administered once every 2 weeks for 4 doses at a lower dose, then once every 4 weeks for 14 doses at a higher dose by intravenous infusion (IV). Among the 32 heavily-pretreated patients, four patients, one of whom was diagnosed with MB, achieved stable disease, which lasted at least 6-months post-NIVO therapy. The “go boundary” was reached for the disease control rate for the patients in this cohort; thus, the number of heavily-pretreated patients will now reach total accrual, and the study will continue to evaluate the survival benefits for NIVO therapy in adult patients with MB. Results of this continued study arm have not been published.

Similarly, in NCT02359565, the Pediatric Brain Tumor Consortium (PBTC) sought to identify the safety and preliminary efficacy of PB in pediatric patients with recurrent or refractory MB and ependymoma (EP) (99). 13 patients with MB were treated with PB every 3 weeks. The median PFS in MB patients trended longer than the ependymoma cohort, but both were less than 2 months. The safety profile of PB demonstrated no dose-limiting toxicities. In their most recent abstract, the authors preliminarily concluded that monotherapy immune checkpoint inhibitors are well tolerated but may not significantly prolong survival in children with recurrent or refractory MB. These results are similarly limited by small sample size and lack of statistical significance. Final study results with correlative biology studies including any subgroup analysis have yet to be published.

CheckMate 908 (NCT3130959) evaluated the use of NIVO with or without anti-CTLA-4 monoclonal antibody, Ipilimumab, in pediatric patients with CNS malignancies including MB (98). Ipilimumab (IPI) is a fully human immunoglobulin IgG1 monoclonal antibody that targets CTLA-4 to sterically inhibit its interaction with its binding partner CD80/86 (100). By inhibiting this interaction, T-cell activation becomes disinhibited (100). IPI was the first FDA approved immune checkpoint inhibitor and is currently approved for use in metastatic melanoma (115) and metastatic or unresectable hepatocellular carcinoma (116). In this study, refractory and recurrent pediatric MB patients either received NIVO alone or in combination with IPI (98). Molecular analysis revealed genetic alterations in *MYC/MYCN, PTCH1, CTNNB1*, and *TP53*, among others. Of the cohort which received NIVO alone, most tumors were classified as either Group 4 or unknown subgroup. Among those who received NIVO with IPI, most patients had unknown subgroup status, and two patients had SHH or Group 3-MB. The median overall survival (OS) for NIVO+IPI was longer than the NIVO alone group by 14.8 months, and the median PFS was 1.4 months longer in the combination therapy cohort. Similarly, the combination therapy group had 12-month overall survival of 86.7% in comparison to the NIVO alone cohort (38.9%). The 6-month PFS was 0% for NIVO alone and 20.0% for the combination group. Although these changes were not statistically different, the authors noted that there is a trend towards improved OS, observed with combination therapy of NIVO+IPI. These trends were unique to the MB patient cohort included in the study and independent of PD-L1 positivity in MB tumors. The combination therapy was well-tolerated with no adverse safety profiles. Notably, the median OS of 22.2 months in the NIVO+IPI arm is similar to that of the Children’s Oncology Group study ACNS0821 where OS in the superior arm containing bevacizumab was 19 months (117). This study tested temozolomide and irinotecan with or without bevacizumab in 105 recurrent and refractory MB. Versions of this regimen are commonly used in recurrent MB across North America (118).

### 3.2 New preclinical and clinical developments

In addition to PD-1, B7-H3, and CTLA-4, more immune checkpoints are currently under investigation for their role in the immunosuppressive TME of MB and therapeutic target identification. For example, Lymphocyte Activation Gene 3 (LAG-3) is an immune checkpoint expressed on NK and T cells that, similar to PD-1, is associated with T-cell exhaustion when ligated with binding partner MHC-II, often found on antigen presenting cells (119). LAG-3 has been identified in MB both through gene expression (120) and at the transcript level (121). For example, methyl-CIBERSORT deconvolution techniques were used to correlate the gene expression of *LAG-3* and other immune checkpoint molecules on infiltrating immune cells with different immune signatures across MB subgroups (120). The study revealed that B7-H3, CTLA-4, LAG-3, PSGL-1 and TIM-3 may all be correlated among MB patients (120), yet further investigation is needed to validate these connections. T cell Immunoglobulin and Mucin domain-containing protein 3 (TIM-3) has also been identified as a potential immune checkpoint target associated with T-cell exhaustion. TIM-3 may also be involved in tumor growth and immune evasion (122–125). When TIM-3 binds its ligand galectin-9, cell death is triggered in T cells expressing TIM-3 (123, 126). TIM-3 has been identified on CD4+ and CD8+ T cells in glioma patients, and increased number of TIM-3+ tumor cells has been associated with improved OS (76). Anti-TIM-3 blockade as part of combination therapy demonstrated increased survival in a mouse model of glioblastoma (127). In addition, TIM-3 transcripts were identified in RNA-seq analysis of MB tumors (120). TIM-3 protein expression was also recently found on myeloid cells infiltrating the MB TME in one study (41) and on tumor-infiltrating lymphocytes in another (76). The latter study also found TIM-3 expressed for the first time on MB tumor cells of all subgroups (76).

P-selectin glycoprotein 1 (PSGL-1) is an adhesion molecule primarily expressed on T cells which regulates T-cell migration via binding to selectins (128, 129). However, it has also been shown to negatively regulate T cell function in adaptive immunity and is an emerging inhibitory immune checkpoint (128). Its transcript has also been identified on infiltrating immune cells in human MB by RNA-seq analysis (41, 120). PSGL-1 is also a binding partner for another emerging immune checkpoint, V-domain Ig Suppressor of T-cell Activation (VISTA) (130, 131). VISTA is a novel B7 family immune checkpoint regulator expressed on myeloid cells including macrophages and microglia (132–134). VISTA has been identified as suppressing T-cell activation and promoting myeloid cell transition to a pro-tumoral state (135). VISTA has been explored in both preclinical and clinical models as an immune checkpoint target for mitigating tumor growth in solid tumors (136, 137). Microglia, macrophage-like TAMs, and T-regulatory cells in the TME of a syngeneic mouse model of MYC-driven MB were shown to express VISTA protein (41). Spatial analysis revealed VISTA-expressing myeloid cells localizing to the tumor-cerebellar border (41). Importantly, VISTA was also found to be highly expressed in human MB at the protein level (41). Further study is required to identify VISTA binding partners, the mechanism of VISTA-mediated T-cell inhibition, and the role of immune checkpoint inhibition of VISTA alone or as combination therapy in MB.

CD155 (PVR) has also recently been identified as a novel immune checkpoint. CD155 is a cell surface poliovirus receptor (PVR) which may be present in MB (138). CD155 mRNA levels were shown to be highest in the WNT subgroup, but its presence was not correlated with overall survival (138). Yet, the study demonstrated that in Group 3 recurrence and metastasis, CD155 expression was high, and antibody-mediated blocking of CD155 protein resulted in cell death (138). CD24 has also been identified as a potential immune checkpoint highly expressed in Group 3 MB (121). CD24 has been shown to interact with Siglec-10, an inhibitory receptor, on macrophages to promote immune escape (139). mRNA levels of CD24, which were associated with worse survival, were highest in SHH, Group 3, and Group 4 MB patients (121). Protein level and functional expression of elevated CD24 levels were not confirmed in this study.

## 4 Discussion

Medulloblastoma is the most common embryonal tumor of the CNS, and yet no targeted immunotherapy has been developed to effectively treat pediatric or adult MB patients. Given that MB is classified into four distinct subgroups, developing subgroup-specific therapies that target underlying molecular mechanisms of MB growth, development, and immune evasion may be effective. Further, efforts to mitigate toxicities associated with standard of care therapy, including neurocognitive changes and secondary malignancy, via novel immunotherapies are essential to improve quality of life in pediatric patients.

Checkpoint inhibitors have emerged as an important immunotherapy in the clinical setting for melanoma, hepatocellular carcinoma, and non-small cell lung cancer, among other solid tumors. However, studies including pediatric brain tumor patients, such as children with MB, are limited. To date, only 7 clinical trials and 1 single institution study explore the use of mono- or combination checkpoint therapy in MB patients. In all studies, MB patients were limited to those with recurrent or refractory disease, and the study did not have enough power to determine the influence of molecular subgroup or specific genetic mutations. Prior studies have shown that individual subgroups may have differing levels of PD-L1 expression both at the protein and transcript level, suggesting the potential for differential sensitivity to immune checkpoint inhibition. However, prospective clinical data clarifying this remains lacking. In addition, our understanding of the immunobiology of MB and the nature of its TME have increased significantly in recent years. Yet, it remains unknown whether overcoming immune suppression lies in targeting immune checkpoint molecules in the TME, in the periphery, or in both. The most recent update of the PBTC study of pembrolizumab in recurrent and refractory MB and EP promises that correlative biology studies are ongoing (99). This data may shed additional light onto mechanisms of immune resistance in MB; however, the small study numbers suggest that uncovering statistically meaningful differences between subgroups will be unlikely.

Clinical trials have revealed that treatment with anti-PD-1 monotherapy or in combination with anti-CTLA-4 therapy is well tolerated in recurrent/refractory MB patients. However, the efficacy in this setting seems to be limited. In fact, multiple large-scale retrospective and prospective studies have demonstrated that the current standard of care is failing a group of patients with high-risk molecular features. For example, in a recent large retrospective analysis, it is apparent that patients identified as having canonical, very high-risk, *MYC-*amplified MB are mostly Group 3 MB. These patients were shown to have no survival benefit irrespective of treatment and experienced only an 11% 5-year PFS (140). In addition, *MYCN*-amplified SHH patients also had extremely poor survival in this study, with only a 20% PFS at 5 years (140). These data suggest that patients with these risk factors are not benefitting from current treatments, and there is a need to develop novel therapies targeted for these patient populations. Whether immune checkpoint inhibitors or other immune based therapies will be beneficial to these very high-risk groups or other subsets remains unknown, as none of the trials have been large enough to meaningfully evaluate the influence of specific subgroups or molecular features to therapeutic response. Only through expanded clinical trials with far greater patient numbers could we identify specific subpopulations that might benefit from ICI. Given the lack of effective therapy at recurrence, expanded trials to address these questions should be considered. Ample evidence indicates that oncologists use ICI off-label particularly in combination with radiation therapy, including in brain tumors (141, 142). Expanding clinical trials to determine whether combination of ICI with RT is safe and effective in pediatric brain tumors has likely been tempered by overall disappointing results in adult glioblastoma (GBM). A recent review summarizes mainly ineffective post RT results but noted promising results with neoadjuvant anti-PD-1 (143, 144). Additional preclinical studies in MB could guide timing of ICI with RT and inform expanded combination clinical trials.

There is a paucity of preclinical studies that define the translational relevance of novel immune checkpoints and subgroup-specific TME alterations in both preclinical models and MB patients. A major barrier to the development of novel immune checkpoint therapy is the difficulty of modeling certain subgroups *in vitro* or *in vivo* as well as the rarity of certain subgroups, particularly WNT. Thus, there is need in the field for both novel approaches to preclinical modeling of MB subgroups as well as new preclinical studies with existing models alongside expanded biobanking initiatives to collect MB patient samples. Only through these combined approaches will we determine optimal immune checkpoint targeting, combination strategies, and therapeutic timing intervals to develop effective immuno-oncology strategies in pediatric MB.

## 5 Conclusions

Medulloblastoma poses significant risk in pediatric patients due to treatment side effects and potential for recurrent/refractory disease. Recurrent and refractory disease remains a therapeutic challenge as there are no effective treatment approaches. Clinical trials examining the effectiveness of immune checkpoint inhibition of PD-1, B7-H3, and CTLA-4 are limited for the treatment of pediatric brain cancers, including MB. Novel immunotherapeutic strategies are crucial to explore, and tackling the cold TME of MB may provide hope for MB patients and provide insight on how to unlock the immunotherapeutic potential of other cold tumors.
